# Evaluating phenotype-driven approaches for genetic diagnoses from exomes in a clinical setting

**DOI:** 10.1038/s41598-017-13841-y

**Published:** 2017-10-18

**Authors:** Reuben J. Pengelly, Thahmina Alom, Zijian Zhang, David Hunt, Sarah Ennis, Andrew Collins

**Affiliations:** 1 Genetic Epidemiology and Genomic Informatics, Faculty of Medicine, University of Southampton, Duthie Building, Mailpoint 808, Tremona Road, Southampton, SO16 6YD UK; 2 Wessex Clinical Genetics Service, Level G, Mailpoint 105, Princess Anne Hospital, Coxford Road, Southampton, SO16 5YA UK

## Abstract

Next generation sequencing is transforming clinical medicine and genome research, providing a powerful route to establishing molecular diagnoses for genetic conditions; however, challenges remain given the volume and complexity of genetic variation. A number of methods integrate patient phenotype and genotypic data to prioritise variants as potentially causal. Some methods have a clinical focus while others are more research-oriented. With clinical applications in mind we compare results from alternative methods using 21 exomes for which the disease causal variant has been previously established through traditional clinical evaluation. In this case series we find that the PhenIX program is the most effective, ranking the true causal variant at between 1 and 10 in 85% of these cases. This is a significantly higher proportion than the combined results from five alternative methods tested (*p* = 0.003). The next best method is Exomiser (hiPHIVE), in which the causal variant is ranked 1–10 in 25% of cases. The widely different targets of these methods (more clinical focus, considering known Mendelian genes, in PhenIX, versus gene discovery in Exomiser) is perhaps not fully appreciated but may impact strongly on their utility for molecular diagnosis using clinical exome data.

## Introduction

Next generation sequencing (NGS) of patient genomes is revolutionising research and medical genetics by establishing molecular diagnoses and identifying novel disease: gene relationships. Whole-exome sequencing (WES), which covers only the protein coding sequence of the genome, is particularly cost-effective and has identified many novel disease genes underlying mostly Mendelian and other monogenic conditions. However WES typically recovers ~30,000 variants of which ~10,000 are predicted to result in nonsynonymous changes, alter conserved splice sites, or represent small insertions or deletions (indels)^1^. The variant set includes many that are potentially deleterious and therefore detailed and careful analysis is required to identify the most likely candidate variant(s) which best match the clinical phenotypes.

In order to reduce the complexity of WES data, methods exist to filter variant lists. Filters discard variants which fail to meet a set of criteria based on, for example, the predicted functional impact of the variant through changes to the protein or whether a variant has been observed in a disease-free control data set. Examples of variant-based prediction tools include SIFT (Sorting Intolerant From Tolerant)^2^ and PolyPhen2 (Polymorphism Phenotyping)^3^, which are concerned with the impact of an amino acid substitution on the structure and function of a protein; GERP++ (Genomic Evolutionary Rate Profiling^4^) which is concerned with evolutionary conservation of sites; VAAST 2.0 (Variant Annotation, Analysis Search tool^5^) which incorporates information about phylogenetic conservation and amino acid substitution and CADD (Combined Annotation-Dependent Depletion^6^) which integrates information from various functional annotations into a single score. Further reduction in the number of candidate variants might be achieved through ‘intersection filtering’^7^ which considers whether a significant proportion of individuals with a shared phenotype carry a predicted damaging variant in the same gene and whether such a variant is a strong candidate for disease causality. However, each genome contains ~100 loss-of-function variants and has ~20 genes completely inactivated^8^. Therefore ‘variant based’ methods based only on predicted pathogenicity, combined with intersection filtering, may be insufficient to separate disease mutations from variants with deleterious biochemical effects which are not related to the disease in question. The difficulty is exemplified by the recent whole genome sequencing of 217 Mendelian disease cases with a broad range of disorders for which disease causal variants were, after comprehensive analysis, confirmed in only 34% of cases^9^. The development and implementation of more powerful strategies which can accelerate the establishment of molecular diagnoses is pressing. Such strategies underlie successful interpretation of cases from the UK 100,000 genomes project (https://www.genomicsengland.co.uk/the-100000-genomes-project/) which is applying NGS to transform patient diagnosis and treatment and rare disease (along with cancer and infectious disease).

Given the difficulty in establishing molecular diagnoses, even for Mendelian forms of disease, a number of tools have been developed which are designed to determine or support the identification of causal variants (Table 1). These methods integrate diverse database information including, for example, phenotypic ontologies, variant pathogenicity scores, insights from model organisms and protein:protein interaction data, with patient phenotypic and genotypic NGS data. To evaluate the utility of these tools for establishing clinical molecular diagnoses we compare results from a range of methods through rank positions for the causal variants in a panel of clinical exomes which have firmly established molecular diagnoses. The tools produce ranked lists of variants but do not report exclusions (i.e. where the causal variant is not within the NGS data file). We compare methods through the ranked position of the causal variant in each case, in particular where a method achieves a rank of 1 for the causal variant or the variant is ranked in the range 1–10. The cases chosen (Supplementary Table 1) form part of a clinical service evaluation of routine NGS diagnostic testing and might be considered representative of cases encountered in a clinical genetics environment.

**Table 1:** Some phenotype-based variant prediction tools.

Tool	Concept	Authors benchmarks	References and software
**Exomiser (hiPHIVE)** (human/interactome-PHenotypic Interpretation of Variants in Exomes)	Integrated phenotypic and interactome analysis using model organisms (mouse, zebrafish) and human clinical data along with protein-protein interaction network data. Focussed on finding new disease genes.	Known disease-gene associations the top hit in 97 % of simulated exomes.	^1,29,30^ http://www.sanger.ac.uk/science/tools/exomiser
**eXtasy**	Integrates predicted impact of variants with haploinsufficiency and phenotype-specific gene prioritisation. Uses random forest learning trained on the Human Gene Mutation Database (HGMD^16^)	Outperforms classical deleteriousness scores (PolyPhen, SIFT, MutationTaster).	^13^ http://extasy.esat.kuleuven.be/
**OMIM Explorer**	Reduces high dimensional phenotypic and genotypic data using semantic similarity and multidimensional scaling. Interface can be used to convert clinical notes to HPO terms.	Clinical variants given median rank of 2, causal variants in top 1% of candidates (47 cases). Outperformed Phen-Gen, eXtasy, and Exomiser (hiPHIVE) for clinical variants.	^17^ http://omimexplorer.research.bcm.edu:3838/omim_explorer/
**OVA** “Ontology Variant Analysis”	Integrates human and model organism phenotypes, functional annotations, curated pathways, cellular localizations and anatomical terms using supervised learning. Exploits multiple ontologies and experimental interaction data^23^.	Outperformed ExomeWalker^31^ in benchmarking with 150 exomes. True disease gene ranked first in 20% on cases.	^18^ http://dna2.leeds.ac.uk:8080/OVA/index.jsp
**Phen-Gen**	Semantic matching of symptoms against disorder database following Phenomizer^14^. Functionally related genes recognised through random walk algorithm. Variants classified using conservation and predicted functionality scores. Phenotypic and genotypic evidence combined in Bayesian framework.	Causal coding variants ranked first in 88% of cases (simulation) and in 8 of 11 patient samples. Outperformed VAAST, eXtasy and Phevor by 13–58% and PHIVE by 13–16%.	^24^ http://phen-gen.org/
**PhenIX** (Phenotypic interpretation of eXomes)	Interrogates only known Mendelian genes and uses semantic similarity matching in Phenomizer^14^. Uses MutationTaster, Polyphen2 and SIFT to predict pathogenicity.	Tests on 52 patient samples with known mutations correct gene achieved mean rank of 2.1	^11^ http://compbio.charite.de/PhenIX/
**Phevor** “Phenotype driven variant ontological re-ranking tool”	Uses ontologies to re-prioritise candidates identified by other variant prioritisation tools such as SIFT, PhastCons and VAAST to identify alleles not previously linked to disease.	Improved performance of tools such as SIFT and VAAST.	^26^ http://weatherby.genetics.utah.edu/cgi-bin/Phevor/PhevorWeb.html

### Overview of current tools

We consider tools which integrate patient phenotypic information (usually represented in the form of Human Phenotype Ontology, HPO terms; http://human-phenotype-ontology.github.io/about.html) with NGS-derived genotypic data in the form of a VCF file^10^. Several of the tools are relatively easy to use through online web servers where HPO terms and VCF files containing patient exome data may be uploaded (Table 1). The methods may have a primarily clinical focus, in which known disease genes are targeted, or have a gene discovery emphasis in which novel genes, showing some relationship to known disease associations, are highlighted. The methods include **PhenIX** ^11^ which ranks candidate genes in NGS data with a focus on known disease-associated Mendelian genes. Ranking is based on integration of predicted variant pathogenicity with phenotypic similarity of diseases associated with these genes. **Exomiser (hiPHIVE)** ^1^ uses the same software framework but also includes multi-species (human, Zebrafish, mouse) ontologies and protein-protein interaction network data. It has a gene discovery focus employing random-walk analyses of multi-species protein interaction networks. Human data come from OMIM and Orphanet^12^ and the human phenotype comparison considers known disease-gene associations while integration of mouse and zebrafish data targets novel candidate genes. Where genes have no known phenotype associations a random-walk-with-restart algorithm scores proximity to other genes in protein-protein association networks which are implicated in patient phenotypes.

**eXtasy** ^13^ employs genomic data fusion to quantify the deleteriousness of nonsynonymous variants which are prioritised dependent on disease phenotypes. eXtasy evaluates patient data against ten measures of variant deleteriousness and a haploinsufficiency prediction score for given gene. The gene prioritisation approach scores genes with mutations according to their similarity with known disease genes. Disease genes previously associated with a HPO term are identified using the Phenomizer algorithm^14^. Genes containing variants are scored for similarity with this set of genes using Endeavour^15^, which recognises the high proportion of shared annotations in gene ontology databases. Random Forest learning is used for data integration with the model trained on the Human Gene Mutation Database^16^ compared to (non-disease) control datasets of common polymorphisms and rare variants.

**OMIM Explorer** ^17^ is strongly focussed towards clinical diagnostics by applying transitive prioritization which links phenotypes to variants through medically recognised intermediates. The tool quantifies semantic similarity to compare patient phenotypes with known diseases or syndromes using OMIM as a basis for calculations. Semantic similarity scores and HPO annotations are used to identify similarities of an input query to the set of OMIM-described diseases defined by HPO phenotypes. The interactive user interface guides user input to gradually improve the diagnostic process. Innovative features include an interface for translating clinical notes into HPO terms.

**OVA** ^18^ considers genotype and predicted effect on protein sequence to reduce the number of potential candidate variants. OVA firstly excludes likely benign variants (such as synonymous and intronic variants) and then evaluates remaining variants against a multi-ontology annotation. Different ontologies are considered which integrate human and model organism data including: Gene Ontology^19^, HPO^14^, Uberon^20^, Disease Ontology^21^ and The Pathway Ontology^22^. Experimental interaction data from mentha^23^ are also considered. For scoring semantic similarity the query phenotypic descriptors and variant data are evaluated against known phenotype-genotype associations, phenotypes and links across ontologies with the target being the prioritisation of known and novel disease genes. Gene scores are optimised using a Random Forest model to classify each candidate gene and obtain final ranks for candidate genes.

**Phen-Gen** ^24^ predicts the damaging impact of coding mutations (nonsynonymous, splice site, and indels) enabling a quantitative comparison between them. Phen-Gen determines potential disease impacts at a locus level (including consideration of non-coding variation) using evolutionary conservation, ENCODE predictions^25^, and proximity to coding sequence. Phenomizer is used for matching patient HPO terms to known disease-gene associations. Novel candidate genes are assessed as functionally related genes using a random-walk-with-restart algorithm searching gene interaction networks. A Bayesian approach is used to evaluate deleterious variants in the exome to known disease-gene associations.

**Phevor** ^26^ integrates phenotype, gene function, and disease information with genomic data targeting both known variation and disease causing alleles not previously implicated in disease. Phevor combines data from biomedical ontologies with variant prioritization scores. The tool propagates information across and between ontologies to re-prioritize potentially damaging variants given gene function and disease, and phenotype knowledge. Outputs from the NGS annotation tools ANNOVAR^27^ and VAAST^5^ are used to rank exome variants. Input patient phenotypes are mapped against a series of ontologies, such as HPO and the Mammalian Phenotype Ontology^28^, to produce a list of genes known to be associated with these terms. In effect entries in different ontologies are brought together through different annotations of the same gene. Each gene receives a score which is combined with the variant annotation data to produce a final rank.

## Results

We examined a total of 21 clinical exomes. In the case of Patient 6, with ‘epileptic encephalopathy, early infantile, 4’ secondary to a mutation in the *ARX* gene, the known causal variant was not captured by the TruSight One panel, and was therefore not present in the genotype data for this individual. We have therefore excluded this case from the comparison of methods.

**Figure 1:**
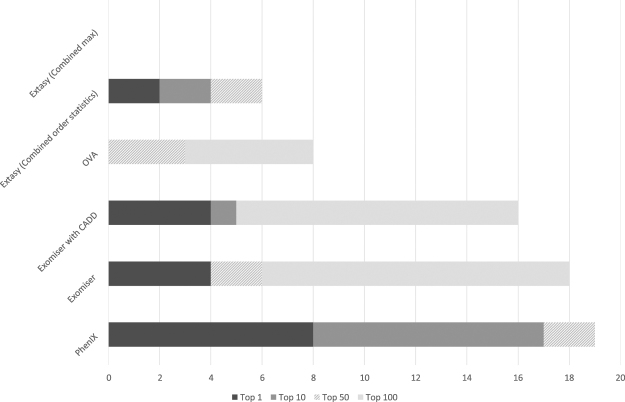
Ranks for causal variants by category. Chart showing the number of cases in different rank classes for each method.

**Figure 2:**
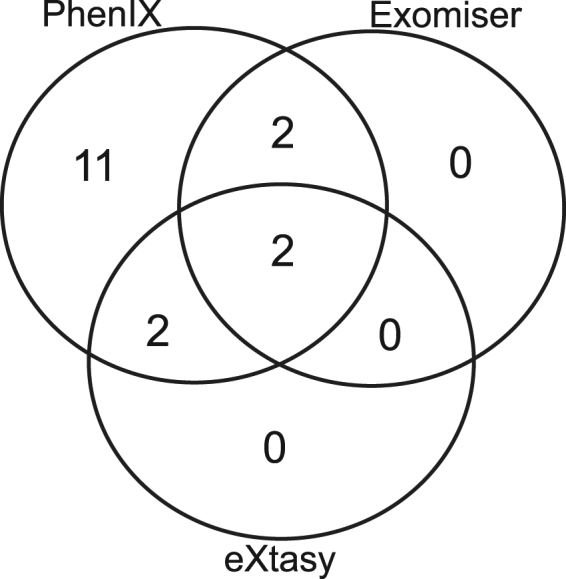
Intersection of pathogenic variants being ranked within the top 10 between software.

Table 2 shows the rank position of the known causal mutation in the set of variants scored by each method. The known pathogenic variant was correctly assigned a rank of 1 in 40% of cases by PhenIX (Table 2, Figs 1 and 2), 20% of cases by Exomiser and 10% of cases by eXtasy (using combined order statistics). OVA and eXtasy (using the maximum score) did not identify the correct variant as rank 1 in any case. Considering the identification of the correct causal variant within a rank of 1–10 the proportion of cases resolved by PhenIX rises to 85% but the proportion remains at 20% for Exomiser and increases slightly to 25% using Exomiser with CADD scores. eXtasy, with combined order statistics, identifies the causal variant with rank 1–10 in 20% of cases. PhenIX places the known causal variant at rank = 1 in 8 cases whereas the five other methods combined (Table 2) identify the known variant at rank = 1 in 5 cases, however, this difference is not significant (*p* = 0.50, by Fisher’s exact test). Considering the placement of the known causal variant as rank = 1–10, PhenIX achieves this in 17 cases whereas taking the highest rank achieved by any method from the set of five other methods ranks the causal variant as 1–10 for 7 cases (*p* = 0.003).

**Table 2:** Rank positions of causal variants by method.

**Patient**	**Gene**	**Diagnosis**	**Rank**					
			**PhenIX**	**Exomiser**	**Exomiser with CADD**	**OVA**	**eXtasy (order statistics)**	**eXtasy (combined max)**
1	*ARID1B*	COFFIN-SIRIS SYNDROME	2	95	132	1037	6013	6184
2	*KCNQ2*	EPILEPTIC ENCEPHALOPATHY	1	85	104	—	1458	8508
3	*SGCE*	MYOCLONIC DYSTONIA	7	—	—	—	239	9304
4	*MED13L*	MENTAL RETARDATION, AUTOSOMAL RECESSIVE 15	106	14	10	1004	2230	4511
5	*RYR1*	CONGENITAL FIBER-TYPE DISPROPORTION MYOPATHY	1	68	85	74	422	8624
6	*ARX*	EPILEPTIC ENCEPHALOPATHY, EARLY INFANTILE, 4	—	—	—	—	—	—
7	*SACS*	SPASTIC ATAXIA, CHARLEVOIX-SAGUENAY TYPE	3	89	77	308	3264	5032
8	*UBE3A*	ANGELMAN SYNDROME	12	74	77	—	178	8728
9	*PTEN*	PTEN HAMARTOMA TUMOR SYNDROME	1	1	1	—	126	8822
10	*DYNC1H1*	SPINAL MUSCULAR ATROPHY, LOWER EXTREMITY, AUTOSOMAL DOMINANT	10	85	86	20	1759	4687
11	*SCN1A*	DRAVET SYNDROME	2	27	53	72	250	8188
12	*TCOF1*	TREACHER COLLINS SYNDROME 3	9	99	92	45	259	8858
13	*OTX2*	MICROPHTHALMIA, ISOLATED 1	5	60	70	73	—	—
14	*EHMT1*	KLEEFSTRA SYNDROME	10	88	95	—	—	—
15	*EFNB1*	CRANIOFRONTONASAL SYNDROME	1	1	1	—	254	8997
16	*HRAS*	COSTELLO SYNDROME	7	1	1	52	1	9328
17	*PTPN11*	NOONAN SYNDROME 6	1	82	83	—	1	9328
18	*EIF2B1*	LEUKOENCEPHALOPATHY WITH VANISHING WHITE MATTER; VWM	11	—	144	—	30	9216
19	*FGFR3*	MUENKE SYNDROME	1	1	1	50	7	9281
20	*POLG*	ALPERS SYNDROME	1	89	98	402	14	8876
21	*COMP*	PSEUDOACHONDROPLASIA	1	78	90	53	10	9310

‘—’ – not ranked.

For eXtasy the superiority of the combined order statistics over the maximum score is clear (Table 2). The performance of eXtasy using combined order statistics might have improved if a complete set of HPO terms could have been used.

The superiority of PhenIX for this small case series of clinical exomes is clear although it is worth noting that improved prioritisation was achieved by alternative methods for two of the cases. For the mental retardation, autosomal recesive 15 case involving the gene *MED13L* (patient 4) PhenIX only achieves a rank of 106 for the causal variant compared to a much improved ranking of 10 using Exomiser with CADD. Although it is not possible to draw firm conclusions from one case it is conceivable that in, for example, mental retardation phenotypes where there is extreme phenotypic and genotypic heterogeneity, the integrated phenotypic and interactome analysis provided by Exomiser is more powerful. The other case where there is apparent superiority over PhenIX is for Costello syndrome (patient 16) for which the causal variant in *HRAS* achieves a rank of 7 under PhenIX but ranked 1 by Exomiser and eXtasy using combined order statistics. The reason for this difference is not understood.

## Discussion

Matching by semantic similarity of patient phenotypes with resources such as the Online Mendelian Inheritance in Man (OMIM) disease catalogue is widely employed. A straightforward analysis strategy might filter variant lists by limiting the search for causal variants to genes already known to contain variants associated with a set of phenotypes, for example using lists of genes generated from OMIM. This seems most likely to be effective for conditions with more limited phenotypic and genotypic heterogeneity but, in other cases, using tools such as PhenIX which allows for phenotypic ambiguity through distance measures in the HPO network, as opposed to using semantic absolutes using OMIM, might be advantageous.

Tools which integrate knowledge of existing clinical phenotype and genotype relationships might give misleading results where these relationships are poorly understood. James *et al*.^17^, argue that the procedure employed in PhenIX (in which phenotypes are collapsed across the diseases to which a gene’s variants have been associated), can result in overestimation and underestimation of semantic similarity matches of candidate genes to patient phenotypes and limited reporting of ruled-out diseases from further consideration. There is therefore a risk of incorrect phenotypic interpretation given the limitations of current knowledge and over-reliance on this form of matching. However, as we have shown here for cases in a clinical setting, tools which have a gene discovery rather than diagnostic emphasis may give misleading results.

Most tools (eXtasy, Phevor, Phen-Gen, OVA and Exomiser hiPHIVE) integrate human and non-human genomic data which underlies their gene discovery focus. Our analyses, which utilise clinical exome data with known molecular causes, suggest that these tools may not reliably identify known disease:gene relationships. The most striking example is in the comparison of PhenIX and Exomiser (hiPHIVE) which share the same software framework but have widely differing performance (ranking the causal variant 1–10, in 17 cases by PhenIX, compared to 5 cases by Exomiser, *p* = 0.0003). This comparison suggests that the integration of model organism data (as in Exomiser) may be less useful in prioritising established human phenotype: genotype relationships which underlie many clinical genetics applications. However, where there is high phenotypic and genotypic heterogeneity, such as in the case of mental retardation phenotypes, tools which encompass a wider range of predictors may be more useful.

Beyond the questions of diagnostic accuracy, there are also other potential factors which would need to be considered prior to the implementation of these tools in a diagnostic setting. Amenability to high throughput use, and ability to integrate with existing software used would greatly reduce the ‘hands on’ time required for using these tools, as well as reducing the potential for user input error. Furthermore, care must be taken regarding data protection. Tools which provide only a website to which patient data is uploaded (for instance OMIMExplorer) will likely raise more concerns than a tool which can be run locally without data leaving the lab (such as the Exomiser software package).

The use of these software tools will obviously fail to correctly identify the pathogenic variant in cases where the pathogenic variant is not present in the sequencing data (as seen in patient 6 with a pathogenic *ARX* variant). Some consideration should also be given to the use of *a priori* candidate gene sets identified using HPO terms (for example by using the Phenomizer platform^14^). Here, candidate genes worthy of sequencing may be identified and this information can impact the choice of panel for the planned sequencing experiment.

It must be noted that this investigation considers only a small sample size, although they represent well characterised clinical cases. Although a total of 20 exomes contribute to the final analyses, it is noteworthy that the statistical superiority of PhenIX in these data has been demonstrated. It is likely that these data are not representative of the substantial variety of exome samples that will be seen in clinical practice, though they do represent an unbiased selection of exomes which were clinically resolvable through traditional genetic investigations. Whilst resolving clinically tractable exomes is perhaps not the area for which these tools offer the biggest gain, they have the potential to help streamline diagnostic processes if used routinely for diagnostic applications. It is therefore important to understand situations in which some of the tools may be sub-optimal. We have shown that this may be the case with hiPHIVE for clinically ‘simple’ cases, and further work is required to confirm this evidence to inform clinical practice as NGS and HPO analyses become increasingly mainstream.

## Materials and Methods

We consider 21 exome samples collected during a regional clinical exome service evaluation project in the UK. These cases have a previously established, clinically confirmed molecular diagnosis determined through traditional testing. Phenotypes from each case were described through comprehensive sets of HPO terms (Supplementary Table 1); HPO terms were selected based upon review of the clinical notes, identifying unambiguous physical features, as well as those reported by multiple clinicians. Samples were sequenced following capture using the TruSight One sequencing panel (Illumina, San Diego, CA, USA). The TruSight One panel captures the exonic regions of 4,813 genes that are known to be implicated in the development of human disease.

For the tools we were able to compare we retained default parameters throughout and used the same HPO terms and VCF files as input in each case, with the following tool-specific differences: eXtasy could not utilise all current HPO terms because its internally held database of HPO terms has not been fully updated since the original publication of the eXtasy program. We consider two alternative statistics for the eXtasy software. Because each variant may be associated with different phenotypes eXtasy can report a maximum score (‘combined max’) across phenotypes^13^ and, alternatively, it may report Order Statistics (‘combined order statistics’)^15^ which combines ranking from separate data sources effectively reducing to a combined rank across all separate ranks. PhenIX was run utilising the available web server (http://compbio.charite.de/PhenIX/), whilst hiPhive was run using the downloaded Exomiser package. For hiPHIVE and PhenIX, we specified a 0.1% allele frequency cutoff. Exomiser (hiPHIVE) does not include CADD scores as a default but has the option to include them if downloaded locally. We compare both the default program and the program with the addition of CADD scores. We scored the rank position determined by each method tested for the known causal variant in every case (Table 2, Fig. 1).

This research was performed in accordance with the relevant guidelines for research within the National Health Service.

### Data availability

We are unable to make the genomic data on which these analyses are based available.

## Electronic supplementary material

Supplementary materials

## References

[1] Robinson PN, Köhler S, Oellrich A (2014). Improved exome prioritization of disease genes through cross-species phenotype comparison. Genome research.

[2] Ng PC, Henikoff S (2003). SIFT: Predicting amino acid changes that affect protein function. Nucleic acids research.

[3] Adzhubei, I., Jordan, D. M. & Sunyaev, S. R. Predicting functional effect of human missense mutations using PolyPhen2. *Current protocols in human genetics* 7–20 (2013). 10.1002/0471142905.hg0720s76 PMC4480630 23315928

[4] Davydov EV, Goode DL, Sirota M (2010). Identifying a high fraction of the human genome to be under selective constraint using GERP++. PLoS Comput Biol..

[5] Hu H, Huff CD, Moore B (2013). VAAST 2.0: Improved variant classification and disease gene identification using a conservation controlled amino acid substitution matrix. Genetic epidemiology.

[6] Kircher M, Witten DM, Jain P (2014). A general framework for estimating the relative pathogenicity of human genetic variants. Nature genetics.

[7] Dand N, Schulz R, Weale ME (2015). Network-Informed Gene Ranking Tackles Genetic Heterogeneity in Exome Sequencing Studies of Monogenic Disease. Human mutation.

[8] MacArthur, D. G., Balasubramanian, S., Frankish, A. *et al*. A systematic survey of loss-of-function variants in human protein-coding genes. *Science* **335**(6070), 823–828 (2012). 10.1126/science.1215040 PMC3299548 22344438

[9] Taylor JC, Martin HC, Lise S (2015). Factors influencing success of clinical genome sequencing across a broad spectrum of disorders. Nature genetics.

[10] Danecek P, Auton A, Abecasis G (2011). The variant call format and VCFtools. Bioinformatics.

[11] Zemojtel T, Köhler S, Mackenroth L (2014). Effective diagnosis of genetic disease by computational phenotype analysis of the disease-associated genome. Science translational medicine.

[12] Rath A, Olry A, Dhombres F (2012). Representation of rare diseases in health information systems: the Orphanet approach to serve a wide range of end users. Human Mutation.

[13] Sifrim A, Popovic D, Tranchevent LC (2013). eXtasy: variant prioritization by genomic data fusion. Nature methods.

[14] Köhler S, Schulz MH, Krawitz P (2009). Clinical diagnostics in human genetics with semantic similarity searches in ontologies. The American Journal of Human Genetics.

[15] Aerts S, Lambrechts D, Maity S (2006). Gene prioritization through genomic data fusion. Nature biotechnology.

[16] Stenson, P. D., Ball, E. V., Mort, M. *et al*. The Human Gene Mutation Database (HGMD) and its exploitation in the fields of personalized genomics and molecular evolution. *Current protocols in bioinformatics* 1–3 (2012). 10.1002/0471250953.bi0113s39 22948725

[17] James RA, Campbell IM, Chen ES (2016). A visual and curatorial approach to clinical variant prioritization and disease gene discovery in genome-wide diagnostics. Genome Medicine.

[18] Antanaviciute, A., Watson, C. M., Harrison, S. M. *et al*. OVA: integrating molecular and physical phenotype data from multiple biomedical domain ontologies with variant filtering for enhanced variant prioritization. *Bioinformatics* btv473 (2015). 10.1093/bioinformatics/btv473 PMC4653395 26272982

[19] Ashburner M, Ball CA, Blake JA (2000). Gene ontology: tool for the unification of biology. The Gene Ontology Consortium. Nature Genetics.

[20] Mungall CJ, Torniai C, Gkoutos GV (2012). Uberon, an integrative multi-species anatomy ontology. Genome Biology.

[21] Kibbe WA, Arze C, Felix V (2015). Disease Ontology 2015 update: an expanded and updated database of human diseases for linking biomedical knowledge through disease data. Nucleic Acids Research.

[22] Petri V, Jayaraman P, Tutaj M (2014). The pathway ontology - updates and applications. J. Biomed. Semantics.

[23] Calderone A, Castagnoli L, Cesareni G (2013). mentha: a resource for browsing integrated protein-interaction networks. Nature Methods.

[24] Javed A, Agrawal S, Ng PC (2014). Phen-Gen: combining phenotype and genotype to analyze rare disorders. Nature methods.

[25] ENCODE Project Consortium (2004). The ENCODE (ENCyclopedia of DNA elements) project. Science..

[26] Singleton MV, Guthery SL, Voelkerding KV (2014). Phevor combines multiple biomedical ontologies for accurate identification of disease-causing alleles in single individuals and small nuclear families. The American Journal of Human Genetics.

[27] Wang K, Li M, Hakonarson H (2010). ANNOVAR: functional annotation of genetic variants from high-throughput sequencing data. Nucleic acids research.

[28] Smith CL, Eppig JT (2009). The mammalian phenotype ontology: enabling robust annotation and comparative analysis. Wiley Interdisciplinary Reviews: Systems Biology and Medicine.

[29] Smedley D, Jacobsen JO, Jäger M (2015). Next-generation diagnostics and disease-gene discovery with the Exomiser. Nature protocols.

[30] Haendel MA, Vasilevsky N, Brush M (2015). Disease insights through cross-species phenotype comparisons. Mammalian Genome.

[31] Smedley D, Köhler S, Czeschik JC (2014). Walking the interactome for candidate prioritization in exome sequencing studies of Mendelian diseases. Bioinformatics.

