# Supplementary material for: Evaluating phenotype-driven approaches for genetic diagnoses from exomes in a clinical setting
Source: Sci Rep. 2017 Oct 18;7:13509. doi: 10.1038/s41598-017-13841-y (PMC5647373; doi:10.1038/s41598-017-13841-y)
Supplement: Supplementary file 1 — Supplementary materials [file 41598_2017_13841_MOESM1_ESM.pdf]

Supplementary material to: Evaluating phenotype-driven approaches for genetic diagnoses from exomes in a clinical setting

Reuben J. Pengelly[1*], Thahmina Alom[1], Zijian Zhang[1], David Hunt[2], Sarah Ennis[1], Andrew Collins[1]

1. Genetic Epidemiology and Genomic Informatics,

Faculty of Medicine,

University of Southampton,

Duthie Building (808),

Tremona Road, Southampton, SO16 6YD, UK.

Tel: 44(0)2381204424; Fax: 44(0)2381204264.

Email: R.J.Pengelly@soton.ac.uk

2. Wessex Clinical Genetics Service

Level G, Mailpoint 105

Princess Anne Hospital

Coxford Road

Southampton

SO16 5YA

*Corresponding author

Supplementary Table 1: HPO terms used as input for each of the patients

Patient	HPO ID	Label
**1**	HP:0011968	Feeding difficulties
	HP:0001249	Intellectual disability
	HP:0001028	Hemangioma
	HP:0000964	Eczema
	HP:0001388	Joint laxity
	HP:0008398	Hypoplastic fifth fingernail
	HP:0000750	Delayed speech and language development
	HP:0001007	Hirsutism
	HP:0000400	Macrotia
	HP:0001558	Decreased fetal movement
	HP:0000218	High palate
	HP:0002650	Scoliosis
	HP:0000347	Micrognathia
**2**	HP:0003808	Abnormal muscle tone
	HP:0001344	Absent speech
	HP:0002305	Athetosis
	HP:0001249	Intellectual disability
	HP:0012450	Chronic constipation
	HP:0001250	Seizures
	HP:0004322	Short stature
	HP:0002307	Drooling
	HP:0000486	Strabismus
	HP:0008872	Feeding difficulties in infancy
	HP:0011344	Severe global developmental delay
	HP:0100021	Cerebral palsy
	HP:0004374	Hemiplegia/hemiparesis
**3**	HP:0001249	Intellectual disability
	HP:0002530	Axial dystonia
	HP:0007010	Poor fine motor coordination
	HP:0001336	Myoclonus
	HP:0100543	Cognitive impairment
**4**	HP:0001537	Umbilical hernia
	HP:0000322	Short philtrum
	HP:0000582	Upslanted palpebral fissure
	HP:0001382	Joint hypermobility
	HP:0000717	Autism
	HP:0008398	Hypoplastic fifth fingernail
	HP:0001263	Global developmental delay
	HP:0006863	Severe expressive language delay
	HP:0010864	Intellectual disability,severe

	HP:0005681	Juvenile rheumatoid arthritis
	HP:0000248	Brachycephaly
	HP:0010808	Protruding tongue
	HP:0000664	Synophrys
	HP:0002136	Broad-based gait
	HP:0000316	Hypertelorism
	HP:0000540	Hypermetropia
	HP:0000414	Bulbous nose
	HP:0001182	Tapered finger
**5**	HP:0003273	Hip contracture
	HP:0002058	Myopathic facies
	HP:0001290	Generalized hypotonia
	HP:0006380	Knee flexion contracture
	HP:0001388	Joint laxity
	HP:0000268	Dolichocephaly
	HP:0001488	Bilateral ptosis
	HP:0002194	Delayed gross motor development
	HP:0002355	Difficulty walking
	HP:0001657	Prolonged QT interval
	HP:0002650	Scoliosis
	HP:0003803	Type 1 muscle fiber predominance
	HP:0004347	Weakness of muscles of respiration
	HP:0002783	Recurrent lower respiratory tract infections
**6**	HP:0011344	Severe global developmental delay
	HP:0002069	Generalized tonic-clonic seizures
	HP:0012469	Infantile spasms
	HP:0002521	Hypsarrhythmia
	HP:0007565	Multiple cafe-au-lait spots
	HP:0100814	Blue nevus
**7**	HP:0000640	Gaze-evoked nystagmus
	HP:0002080	Intention tremor
	HP:0002370	Poor coordination
	HP:0001272	Cerebellar atrophy
	HP:0002936	Distal sensory impairment
	HP:0001260	Dysarthria
	HP:0002317	Unsteady gait
	HP:0003390	Sensory axonal neuropathy
	HP:0003487	Babinski sign
**8**	HP:0011344	Severe global developmental delay
	HP:0001601	Laryngomalacia
	HP:0002069	Generalized tonic-clonic seizures
	HP:0008504	Moderate sensorineural hearing impairment
	HP:0002136	Broad-based gait
	HP:0012011	EEG with occipital focal spike waves

	HP:0002395	Lower limb hyperreflexia
	HP:0000253	Progressive microcephaly
	HP:0010845	EEG with generalized slow activity
	HP:0000733	Stereotypic behavior
	HP:0000494	Downslanted palpebral fissures
**9**	HP:0000256	Macrocephaly
	HP:0012721	Venous malformation
	HP:0002019	Constipation
	HP:0000965	Cutis marmorata
	HP:0001622	Premature birth
	HP:0001270	Motor delay
	HP:0002013	Vomiting
	HP:0011471	Gastrostomy tube feeding in infancy
**10**	HP:0002562	Low-set nipples
	HP:0002600	Hyporeflexia of lower limbs
	HP:0000426	Prominent nasal bridge
	HP:0012650	Perisylvian polymicrogyria
	HP:0002987	Elbow flexion contracture
	HP:0000268	Dolichocephaly
	HP:0008780	Congenital bilateral hip dislocation
	HP:0006610	Wide intermamillary distance
	HP:0002355	Difficulty walking
	HP:0005750	Contractures of the joints of the lower limbs
	HP:0006201	Hypermobility of distal interphalangeal joints
	HP:0000218	High palate
	HP:0005850	Congenital talipes calcaneovalgus
	HP:0007002	Motor axonal neuropathy
	HP:0000347	Micrognathia
	HP:0011167	Focal tonic seizures
**11**	HP:0012368	Flat face
	HP:0001251	Ataxia
	HP:0002123	Generalized myoclonic seizures
	HP:0000252	Microcephaly
	HP:0000750	Delayed speech and language development
**12**	HP:0011968	Feeding difficulties
	HP:0000369	Low-set ears
	HP:0008551	Microtia
	HP:0000347	Micrognathia
	HP:0000316	Hypertelorism
	HP:0000494	Downslanted palpebral fissures
	HP:0005439	Maxillozygomatic hypoplasia
**13**	HP:0000528	Anophthalmia
	HP:0007633	Bilateral microphthalmos
	HP:0008551	Microtia

**14**	HP:0001028	Hemangioma
	HP:0001642	Pulmonic stenosis
	HP:0001290	Generalized hypotonia
	HP:0000431	Wide nasal bridge
	HP:0001263	Global developmental delay
	HP:0002643	Neonatal respiratory distress
	HP:0002360	Sleep disturbance
	HP:0000954	Single transverse palmar crease
	HP:0000316	Hypertelorism
	HP:0000286	Epicanthus
	HP:0001631	Atria septal defect
	HP:0002783	Recurrent lower respiratory tract infections
**15**	HP:0011315	Unicoronal synostosis
	HP:0000486	Strabismus
	HP:0002119	Ventriculomegaly
	HP:0000456	Bifid nasal tip
	HP:0001545	Anteriorly placed anus
	HP:0001290	Generalized hypotonia
	HP:0001274	Agenesis of corpus callosum
	HP:0000316	Hypertelorism
	HP:0000540	Hypermetropia
	HP:0001388	Joint laxity
**16**	HP:0001250	Seizures
	HP:0001348	Brisk reflexes
	HP:0001508	Failure to thrive
	HP:0001639	Hypertrophic cardiomyopathy
	HP:0008873	Disproportionate short-limb short stature
	HP:0001357	Plagiocephaly
	HP:0001998	Neonatal hypoglycemia
	HP:0011470	Nasogastric tube feeding in infancy
	HP:0001263	Global developmental delay
	HP:0002643	Neonatal respiratory distress
	HP:0100501	Recurrent bronchiolitis
	HP:0000470	Short neck
	HP:0000280	Coarse facial features
	HP:0001561	Polyhydramnios
	HP:0000954	Single transverse palmar crease
	HP:0011675	Arrhythmia
**17**	HP:0000369	Low-set ears
	HP:0000884	Prominent sternum
	HP:0004309	Ventricular preexcitation
	HP:0001642	Pulmonic stenosis
	HP:0007018	Attention deficit hyperactivity disorder
	HP:0000540	Hypermetropia

	HP:0000028	Cryptorchidism
	HP:0000494	Downslanted palpebral fissures
	HP:0000286	Epicanthus
	HP:0000431	Wide nasal bridge
**18**	HP:0008209	Premature ovarian failure
	HP:0000786	Primary amenorrhea
	HP:0002069	Generalized tonic-clonic seizures
	HP:0002073	Progressive cerebellar ataxia
	HP:0012429	Aplasia/Hypoplasia of the cerebral white matter
	HP:0008639	Gonadal hypoplasia
**19**	HP:0005280	Depressed nasal bridge
	HP:0011318	Bicoronal synostosis
	HP:0002007	Frontal bossing
	HP:0000218	High palate
	HP:0001901	Polycythemia
	HP:0000494	Downslanted palpebral fissures
	HP:0001998	Neonatal hypoglycemia
	HP:0005487	Prominent metopic ridge
**20**	HP:0001488	Bilateral ptosis
	HP:0006834	Developmental stagnation at onset of seizures
	HP:0001251	Ataxia
	HP:0002121	Absence seizures
	HP:0000572	Visual loss
	HP:0001263	Global developmental delay
**21**	HP:0002515	Waddling gait
	HP:0005060	Limited elbow flexion/extension
	HP:0008873	Disproportionate short-limb short stature
	HP:0009882	Short distal phalanx of finger
	HP:0005085	Limited knee flexion/extension
	HP:0001182	Tapered finger
